# The prognostic impact of neutrophil-to-lymphocyte ratio (NLR) and lymphocyte-to-monocyte ratio (LMR) in patients with distal bile duct cancer

**DOI:** 10.1186/s12957-020-01847-2

**Published:** 2020-04-22

**Authors:** Yoji Miyahara, Shida Takashi, Yoshiaki Shimizu, Masayuki Ohtsuka

**Affiliations:** 1 Department of Surgery, Japanese Red Cross Narita Hospital, 90-1 Iida-cho, Narita-shi, Chiba Prefecture 286-0041 Japan; 2 Department of General Surgery, Graduate School of Medicine, Chiba University, 1-8-1 Inohana, Chuo-ku, Chiba-shi, Chiba Prefecture 260-8677 Japan; 3 Shida Clinic, 1970-1-2 Ne, Shiroi-shi, Chiba Prefecture 270-1431 Japan

**Keywords:** NLR, LMR, Distal bile duct cancer, Prognostic maker

## Abstract

**Background:**

A growing body of evidence suggests that inflammatory response markers such as the neutrophil-to-lymphocyte ratio (NLR) and lymphocyte-to-monocyte ratio (LMR) are associated with outcomes of various malignancies. However, no study has reported the prognostic value of NLR and LMR in patients with distal bile duct cancer (DBDC) to date. We investigated the prognostic significance of these inflammatory markers in patients with DBDC who underwent radical resection.

**Methods:**

The study included 40 patients diagnosed with DBDC who underwent pancreaticoduodenectomy at Narita Red Cross Hospital between January 2000 and December 2017. The cutoff values for these markers were determined by receiver operating characteristic curve analysis. Survival curves are estimated for each group in the study considered separately using the Kaplan-Meier method. The association between overall survival (OS) and the NLR, LMR, and other prognostic factors was investigated using log-rank test and multivariate Cox proportional hazards regression analysis.

**Results:**

Corresponding to the point with the maximum combined sensitivity and specificity on the ROC curve, the best cutoff value for NLR and LMR was determined to be 3.14 and 4.55, respectively. Most clinicopathological factors were not associated with the NLR and LMR based on these cutoff values. However, serum albumin levels were associated with both the NLR and LMR (*P* = 0.011 and *P* = 0.023, respectively), and serum carbohydrate antigen 19-9 (CA 19-9) levels were also associated with the LMR (*P* = 0.030). Univariate analysis showed that a high NLR (*P* < 0.001), low LMR (*P* = 0.002), hypoalbuminemia (*P* = 0.004), high serum CA 19-9 levels (*P* = 0.008), and lymph node metastasis (*P* = 0.033) were significantly associated with poor survival rates. Multivariate analysis showed that a high NLR (hazard ratio 5.799, 95% confidence interval 1.188–28.32, *P* = 0.030) and a low LMR (hazard ratio 4.837, 95% confidence interval 1.826–2.331, *P* = 0.025) were independent prognostic factors for OS.

**Conclusion:**

Both NLR and LMR may serve as significant independent preoperative prognostic indicators of disease in patients with DBDC who undergo radical resection.

## Background

Bile duct cancer is rare and represents a diverse anatomical location. Distal bile duct cancer (DBDC) originates from extrahepatic bile ducts just distal to the cystic duct and proximal to the duodenal ampulla. This malignancy accounts for approximately 20–30% of all bile duct cancers [1]. Patients with DBDC treated with pancreaticoduodenectomy, which is the standard procedure performed for radical resection of DBDC, tend to show a relatively poor prognosis. The 5-year survival rate is approximately 40% owing to high recurrence rates associated with this malignancy [2]. Among the aforementioned postoperative prognostic factors, such as lymph node metastasis, histopathological differentiation, and resection status, are widely known to be associated with long-term survival; however, the role of preoperative factors, if any, remains unclear [3].

The neutrophil-to-lymphocyte ratio (NLR) and lymphocyte-to-monocyte ratio (LMR) are well-known systemic inflammatory response markers; several studies have reported that NLR and LMR are important prognostic factors in various cancers [4–8]. NLR and LMR values can be obtained preoperatively from routine blood tests; therefore, they serve as less invasive and cost-effective markers for preoperative evaluation of patients. However, to date, the prognostic significance of NLR and LMR in patients with DBDC who undergo pancreaticoduodenectomy remains unclear.

In this single-center study, we investigated the association between these biomarkers and survival in patients with DBDC who underwent pancreaticoduodenectomy.

## Methods

### Patient selection and diagnosis of distal bile duct cancer

This study included 40 patients diagnosed with DBDC who were treated with pancreaticoduodenectomy at Narita Red Cross Hospital between January 2000 and December 2017. Preoperative diagnosis of DBDC was based on imaging studies, including computed tomography, magnetic resonance imaging, and endoscopic retrograde cholangiopancreatography. The distal bile duct was confined to the area between the origin of the cystic duct and the duodenal ampulla. The American Joint Committee on Cancer (7th edition, 2009) staging system was used to stage the cancer [9]. Patients with cholangitis underwent preoperative endoscopic biliary drainage or percutaneous transhepatic biliary drainage.

### Variables, definitions, and cutoff values

The NLR was calculated by dividing the total neutrophil count (obtained from peripheral blood samples) by the total lymphocyte count, and the LMR was calculated as the ratio of the total number of lymphocytes divided by the total number of monocytes. All blood samples were obtained during routine preoperative evaluation of patients scheduled for elective surgery. Receiver operating characteristic (ROC) curves were plotted to verify the accuracy of these biomarkers as prognostic indicators of overall survival (OS). We selected cutoff values for NLR, LMR, and tumor markers based on ROC curve analysis to predict 5-year survival. Furthermore, using the modified Glasgow Prognostic Score, the cutoff values for low serum albumin and high C-reactive protein (CRP) levels were calculated as 3.5 g/dL and 0.5 mg/dL, respectively [10].

### Statistical analysis

Survival curves are estimated for each group in a study considered separately using the Kaplan-Meier method. The association between the aforementioned prognostic indicators and OS rates was investigated using the log-rank test and Cox proportional hazards regression analysis. The preoperative indicators observed to be statistically significant (*P* < 0.05) in univariate analysis were subjected to multivariate Cox proportional hazards regression analysis. A *P* value < 0.05 was considered statistically significant. All statistical analyses were performed using the JMP Pro software, version 13.2.0 (SAS Institute Inc., Cary, NC).

## Results

### Clinicopathological features

Baseline patient characteristics are described in Table 1. The median age was 70 years (range 48–77 years), and 33 patients (82%) were men. Histopathological examination revealed well, moderately, and poorly differentiated cancers in 8 (20%), 30 (77%), and 2 patients (3%), respectively. T1, T2, T3, and T4 stage cancers were observed in 4 (10%), 6 (15%), 29 (73%), and 1 patient (3%), respectively. Lymph node metastasis was detected in 19 patients (48%), and 29 patients (73%) underwent R0 resection. The median NLR and LMR values were 1.98 (interquartile range [IQR] 1.37–2.54) and 4.75 (IQR 2.93–5.84), respectively.

**Table 1:** Demographic and clinicopathological features

Variables		Total (*n* = 40)
Age (year)	Mean ± SD	70 (66–77)
Gender	Male	33 (82%)
	Female	7 (18%)
Differentiation	Well	8 (20%)
	Moderately	30 (77%)
	Poorly	2 (3%)
T classification	T1	4 (10%)
	T2	6 (15%)
	T3	29 (73%)
	T4	1 (3%)
N classification	N (+)	19 (48%)
	N (−)	21 (52%)
Resection margin	R0	29 (73%)
	R1	8 (20%)
	R2	3 (8%)
alb (g/dL)	Mean ± SD	3.7 (3.3–3.9)
CRP (mg/dL)	Mean ± SD	0.27 (0.1–1.3)
CEA (ng/mL)	Mean ± SD	2.6 (1.9–4.0)
CA19-9 (U/mL)	Mean ± SD	53.9 (10.1–173.5)
WBC (/mm^3^)	Mean ± SD	5800 (4200–7000)
Hb (g/dL)	Mean ± SD	11.6 (10.5–13.1)
plt (10^4^/mm^3^)	Mean ± SD	22.2 (19.4–26.7)
NLR	Mean ± SD	1.98 (1.37–2.54)
LMR	Mean ± SD	4.75 (2.93–5.84)

### Cutoff values for the neutrophil-to-lymphocyte ratio, the lymphocyte-to-monocyte ratio, and tumor markers

**Fig. 1:**
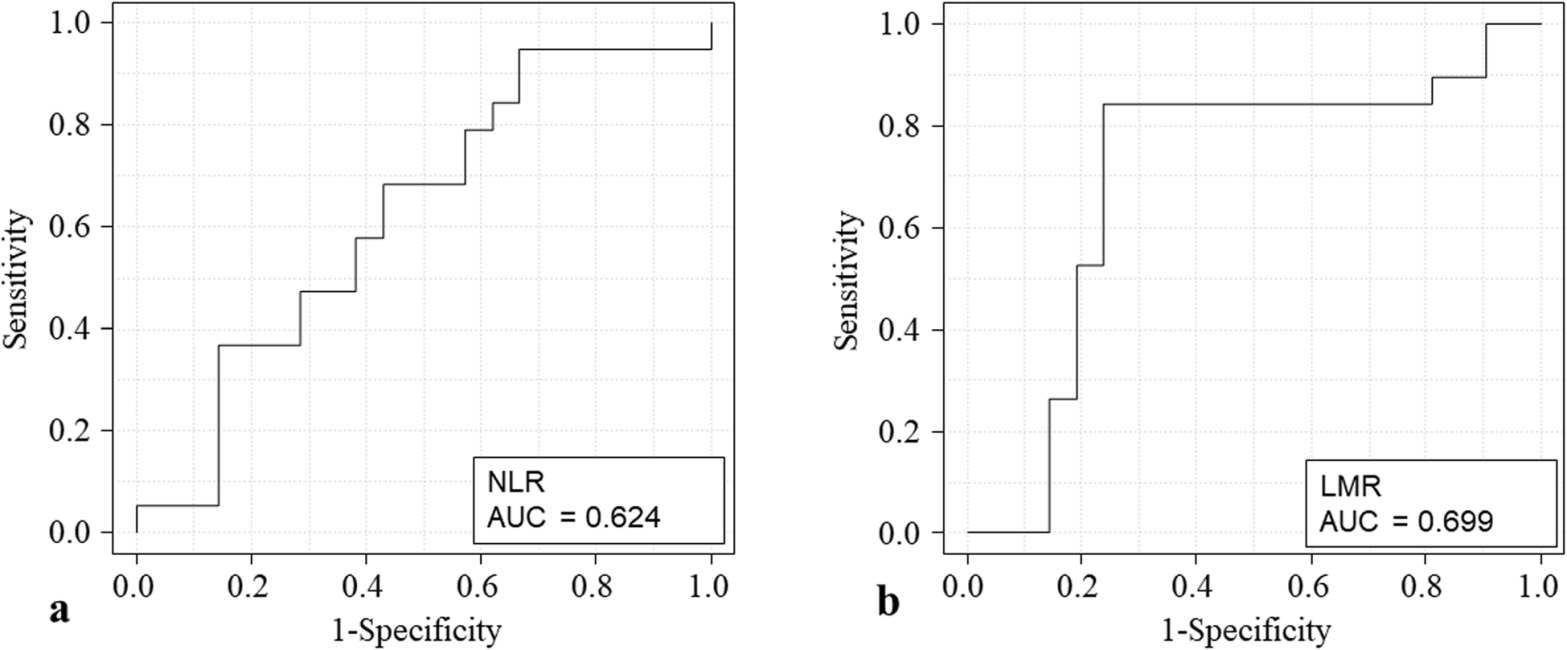
Receiver operating characteristic (ROC) curve analysis for 5 years survival to set cutoff values for neutrophil-to-lymphocyte ratio (NLR) and lymphocyte -to-monocyte ratio (LMR). The cutoff value for NLR and LMR were 3.14 and 4.55, respectively. The area under the curve (AUC) for NLR and LMR was 0.624 and 0.699, respectively

The area under the curve (AUC) for NLR calculated from the ROC curve was 0.640 (95% confidence interval [CI] 0.450–0.850). Corresponding to the point with the maximum combined sensitivity and specificity on the ROC curve, the best cutoff value for NLR was 3.14. Based on this cutoff value, 9 patients (28%) were assigned to the high NLR group. The cutoff value of LMR was determined to be 4.55, and 11 patients (28%) were assigned to the high LMR group. The AUC for LMR was 0.640 (95% CI 0.450–0.850) (Fig. 1). Cutoff values for serum carcinoembryonic antigen (CEA) and carbohydrate antigen 19-9 (CA 19-9) were determined to be 2.4 ng/mL and 60.2 U/mL, respectively. Eleven patients (34%) were classified into the high CEA and 12 patients (38%) into the high CA 19-9 group.

### Collinearity of clinicopathological factors to the neutrophil-to-lymphocyte ratio and lymphocyte-to-monocyte ratio

We validated the association of the clinicopathological factors between these groups (Table 2). Our analysis showed that most clinicopathological factors were not associated with the NLR and LMR. However, serum albumin levels were associated with both the NLR and LMR (*P* = 0.011 and *P* = 0.023, respectively), and serum CA 19-9 levels were also associated with the LMR (*P* = 0.030). Additionally, the collinearity between NLR and LMR was relatively small (Pearson’s correlation coefficient = 0.156).

**Table 2:** The collinearity of clinicopathological factors between groups that divided by NLR and LMR

Factors		NLR			LMR		
		NLR ≥ 3.14	NLR < 3.14	*P* value	LMR ≥ 4.55	LMR < 4.55	*P* value
Age (years)	≥ 65	5	26	0.503	19	12	0.135
	< 65	2	7		3	6	
Gender	Male	6	27	0.645	17	16	0.297
	Female	1	6		5	2	
Differentiation	well differentiated	0	8	0.181	3	5	0.237
	others	7	25		19	13	
T classification	T1/2	2	8	0.572	6	4	0.503
	T3/4	5	25		16	14	
N classification	N (−)	4	17	0.559	14	7	0.203
	N (+)	3	16		8	11	
Resection margin	R0	6	23	0.364	17	12	0.347
	R1 or R2	1	10		5	6	
Albumin (g/dL)	≥ 3.5	1	23	**0.011**	17	7	**0.016**
	< 3.5	6	10		5	11	
CRP (mg/dL)	≥ 0.5	4	12	0.273	6	10	0.068
	< 0.5	3	21		16	8	
CEA (ng/mL)	≥ 2.5	4	17	0.559	12	9	0.512
	< 2.5	3	16		10	9	
CA19-9 (U/mL)	≥ 60.2	5	14	0.164	7	12	**0.030**
	< 60.2	2	19		15	6	

### Association between prognostic indicators and long-term survival on univariate analysis

**Fig. 2:**
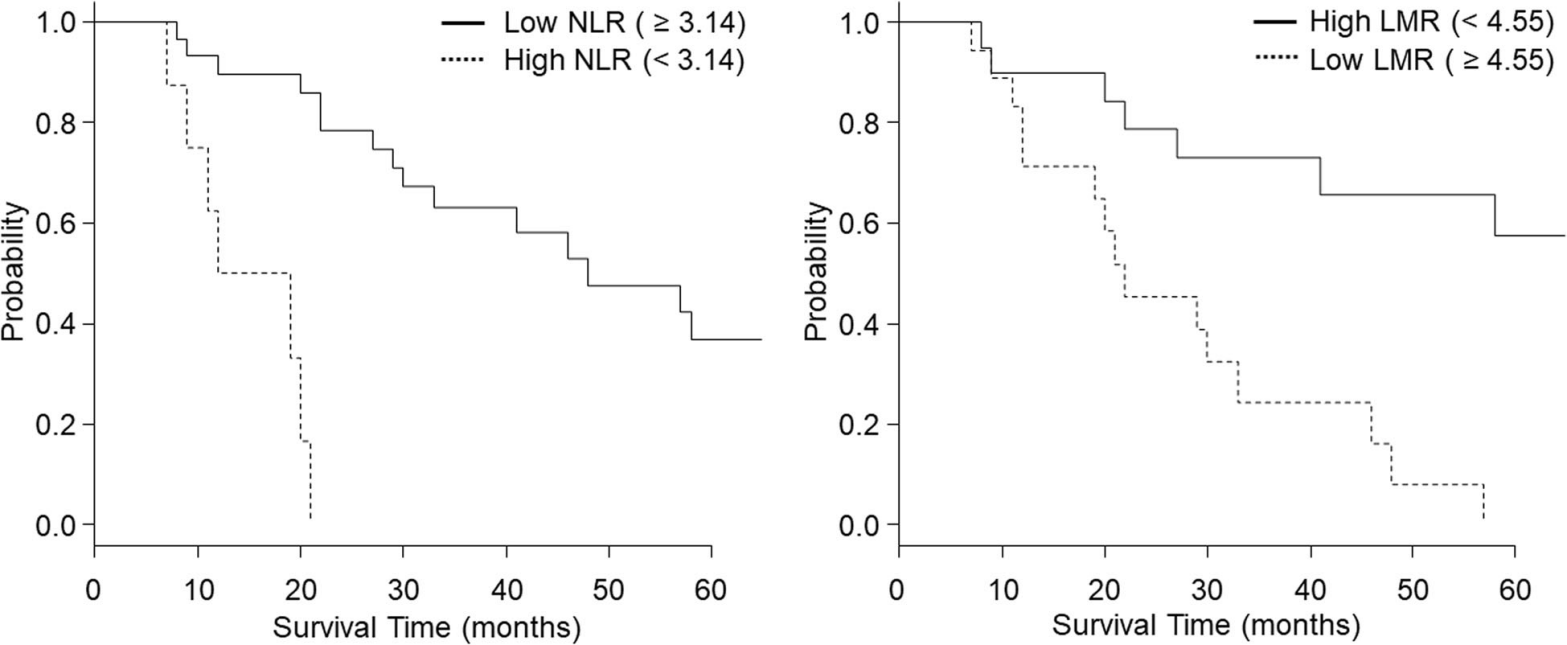
**a** Kaplan-Meier estimates of overall survival (OS) based on neutrophil-to-lymphocyte ratio (NLR) for patient with distal bile duct cancer (log-rank test *P* < 0.001). **b** Kaplan-Meier estimates of OS based on lymphocyte-to-monocyte ratio (LMR) for patient with distal bile duct cancer (log-rank test *P* < 0.001)

The comparison of Kaplan-Meier survival curves using the log-rank test revealed that the NLR and LMR were significantly associated with OS (Fig. 2). The median survival time of the high NLR group was 14.9 months and that of the low NLR group was 65.9 months (*P* < 0.001). The median survival time of the low LMR group was 27.0 months and that of the high LMR group was 82.2 months (*P* < 0.001).

Cox proportional hazards regression analysis was also performed to investigate the association between OS and age, histopathological differentiation, serum albumin, CRP, CEA, and CA 19-9 levels, as well as the NLR and LMR. Univariate analysis showed that hypoalbuminemia (*P* = 0.004), lymph node metastasis (*P* = 0.033), a high NLR (*P* < 0.001), and low LMR (*P* = 0.002) were significantly associated with poor OS. Furthermore, only those preoperative factors that were observed to be statistically significant on univariate analysis were subjected to multivariate analysis using the Cox proportional hazards regression model. Multivariate analysis showed that only a high NLR (hazard ratio 6.133, 95% CI 1.453–25.90, *P* = 0.014) and low LMR (hazard ratio 3.16, 95% CI 1.024–9.751, *P* = 0.045) were independently associated with poor OS (Table 3).

**Table 3:** Univariate and maltivariate analysis of the association between preoperative clinicopathological factors and overall survival in patients with distal bile duct cancer who underwent pancreaticoduodenectomy

Factors		No. (%)	Univariate analysis			Multivariate analysis		
			Hazard ratio	95% CI	*P* value	Hazard ratio	95% CI	*P* value
Age (years)	≥ 65	31 (82%)	0.508	0.2046–1.245	0.138			
	< 65	9 (22%)	(Referent)					
Gender	Male	33 (87%)	0.945	0.352–3.282	0.920			
	Female	7 (13%)	(Referent)					
Differentiation	Well differentiated	8 (20%)	1.243	0.416–3.716	0.697			
	Others	32 (80%)	(Referent)					
T classification	T1/2	10 (25%)	(Referent)	0.6476–5.816	0.236			
	T3/4	30 (75%)	1.941					
N classification	N (−)	21 (53%)	(Referent)	1.078–5.772	**0.033**			
	N (+)	19 (48%)	2.495					
Resection margin	R0	29 (73%)	(Referent)	0.9687–5.581	**0.059**			
	R1 or R2	11 (27%)	2.325					
Albumin (g/dL)	≥ 3.5	24 (60%)	(Referent)	1.521–8.495	**0.004**	1.484	0.537–4.107	0.447
	< 3.5	16 (40%)	3.595					
CRP (mg/dL)	≥ 0.5	16 (40%)	1.220	0.5223–2.85	0.646			
	< 0.5	24 (60%)	(Referent)					
CEA (ng/mL)	≥ 2.5	21 (53%)	1.665	0.7226–3.838	0.231			
	< 2.5	19 (48%)	(Referent)					
CA19-9 (U/mL)	≥ 60.2	19 (48%)	3.165	1.345–7.449	**0.008**	2.614	0.978–6.993	0.056
	< 60.2	21 (53%)	(Referent)					
NLR	≥ 3.14	8 (20%)	11.810	3.296–42.32	**< 0.001**	6.133	1.453–25.900	**0.014**
	< 3.14	32 (80%)	(Referent)					
LMR	≥ 4.55	18 (45%)	(Referent)	1.826–12.81	**0.002**	3.16	1.024–9.751	**0.045**
	< 4.55	22 (55%)	4.837					

In this cohort, 5 patients died of other diseases; stomach cancer: leukemia, acute myocardial infarction (1 patient each), and aspiration pneumonia (2 patients). The mean survival time of these 5 patients was relatively long (36.2 months), suggesting that the effect on survival was not significant. The comparison of Kaplan-Meier survival curves using the log-rank test showed that both NLR and LMR were significantly related to disease-free survival (*P* < 0.001 and *P* = 0.018, respectively).

## Discussion

Our study highlights that preoperative NLR and LMR values were independent predictors of OS in patients with DBDC who underwent pancreaticoduodenectomy. Several studies have reported that preoperative indicators, such as serum albumin and CRP levels, as well as widely recognized tumor markers (CEA and CA 19-9) may also serve as prognostic indicators of biliary tract cancer outcomes [10–14]; however, the NLR and LMR scored over these factors in this study. The prognostic value of these inflammatory biomarkers has been demonstrated in biliary tract cancer [15, 16]. Notably, biliary tract cancer includes intrahepatic cholangiocarcinoma, hilar cholangiocarcinoma, DBDC, and gallbladder adenocarcinoma. These cancers are defined and categorized based on the anatomical sites of involvement of the biliary tree; however, these cancer types vary in their biological behavior. Considering the heterogeneity in biological behavior, which may lead to a variety of immunological reactions, we focused on cancer from a single anatomical origin. Pancreaticoduodenectomy and extrahepatic bile duct resection are used to treat DBDC; however, pancreaticoduodenectomy is the only radical operation for DBDC in the view of lymph node dissection. To eliminate operation bias in this study, we enrolled patients with DBDC who underwent pancreaticoduodenectomy.

The potential mechanisms by which inflammation contributes to cancer progression are as follows: neutrophils inhibit the host immune response to cancer by suppressing cytotoxic immune cells via the secretion of cytokines and chemokines [4] and promote cancer growth and metastasis via their angiogenic action and adherence of circulating tumor cells to the metastatic niche [17]. Although the cause and effect of the association between lymphopenia and poor outcomes remain unclear, lymphocytes are well-known components of cancer immunity, and some studies have reported that the magnitude of the immune response is directly proportional to the number of these cells [18, 19]. Furthermore, myeloid cell infiltration of tumor tissue is considered the key phenomenon associated with cancer immunity [20, 21]. Notably, tumor-associated macrophages (TAMs) derived from monocytes within cancerous tissue promote carcinogenesis by tumor proliferation, angiogenesis, cell invasion, and lymphocyte inhibition [20]. Reportedly, the peripheral monocyte count reflects the density of TAMs in the cancerous tissue [22], and the NLR and LMR reflect the balance of the host immune response and cancer progression. This might explain the role of NLR and LMR as potential prognostic factors. A recent study has reported oncogenetic changes that contribute to an inflammatory microenvironment that promotes tumorigenesis in the presence of inflammatory cells and mediators [23]. Oncogenetic changes activate transcription factors, such as the nuclear factor-κ-B [24], signal transducer and activator of transcription-3, and the hypoxia-inducible factor 1α [25, 26] in tumor cells, all of which favor the production of inflammatory mediators, including cytokines and chemokines [27, 28]. These reactions recruit inflammatory cells including neutrophils and those of myelomonocytic lineage. Therefore, patients with oncogenetic mutations might show a high NLR and low LMR.

Patients with DBDC show poor survival despite treatment. Pancreaticoduodenectomy ensures radical resection of DBDC; however, this operation is one of the most invasive and complicated procedures among all gastrointestinal surgeries. DBDC is a highly invasive tumor, and involvement of surrounding organs can result in postoperative complications that reduce patients’ quality of life or may even be fatal. Therefore, it is important to determine whether operation is necessary for individual patients. The results of the present study highlight the role of NLR and LMR as preoperative prognostic predictors in patients with DBDC. Therefore, in addition to surgery, therapeutic options such as neoadjuvant chemotherapy can be considered in these patients. The efficacy of preoperative and postoperative chemotherapy remains unclear in patients with DBDC [29–31]; however, perioperative chemotherapy may improve prognosis in patients with a high NLR and low LMR.

The limitations of this study include the following: (a) Owing to the rarity of DBDC, this study included a small number of patients. (b) The uncontrolled and retrospective design is a drawback of this study. Despite these limitations, our study is the first to prove that preoperative NLR and LMR serve as independent predictors of OS in patients with DBDC undergoing radical resection. Further accumulation of cases and future cohort studies are warranted to provide a deeper understanding of the mechanism through which a high NLR and low LMR serve as predictors of poor survival in patients with DBDC.

## Conclusion

Both preoperative NLR and LMR were independently associated with survival in patients with DBDC who underwent radical resection. The NLR and LMR can be estimated preoperatively; therefore, these biomarkers can help surgeons in preoperative evaluation of patients.

## Data Availability

The dataset supporting the conclusions of this article is included within the article.

## Abbreviations

NLR: Neutrophil-to-lymphocyte ratio
LMR: Lymphocyte-to-monocyte ratio
DBDC: Distal bile duct cancer
ROC: Receiver operating characteristic
OS: Overall survival
CT: Computed tomography
MRI: Magnetic resonance imaging
ERCP: Endoscopic retrograde cholangiopancreatography
AJCC: The American Joint Committee on Cancer
EBD: Endoscopic biliary drainage
PTBD: Percutaneous transhepatic biliary drainage
mGPS: Modified Glasgow Prognostic Score
CRP: C-reactive protein
SD: Standard deviation
IQR: Interquartile range
AUC: The area under the curve
CI: Confidence interval
